# Retraction: Facile synthesis and electrochemical analysis of TiN-based ZnO nanoparticles as promising cathode materials for asymmetric supercapacitors

**DOI:** 10.1039/d6na90058e

**Published:** 2026-07-31

**Authors:** Junaid Riaz, Jianchun Cao, Yongguo Zhang, Amina Bibi, Xiaolong Zhou

**Affiliations:** a Faculty of Material Science and Engineering, Kunming University of Science and Technology Kunming 650093 China junaidriaz1990@gmail.com nmcjc@163.com kmzxlong@163.com; b Key Laboratory of Advanced Materials of Yunnan Province Kunming Yunnan 650093 PR China; c Department of Physics, Hazara University Mansehra 21300 Pakistan

## Abstract

Retraction of ‘Facile synthesis and electrochemical analysis of TiN-based ZnO nanoparticles as promising cathode materials for asymmetric supercapacitors’ by Junaid Riaz *et al.*, *Nanoscale Adv.*, 2024, **6**, 5145–5157, https://doi.org/10.1039/d4na00372a.

The Royal Society of Chemistry hereby wholly retracts this *Nanoscale Advances* article due to concerns with the reliability of the FESEM data in Fig. 3 and the EDX data in Fig. 4.

The FESEM data in Fig. 3e was first published in ref. 1 as another material.

In the EDX data in Fig. 4b, there are unexpected repeating patterns. In addition, sections of the data for Fig. 4a and c, and Fig. 4b and c have unexpected similarity.

The authors provided the raw data; however, the raw EDX data contained signs of manipulation.

Given the significance of these concerns, the findings presented in this paper are no longer reliable.

The authors did not respond to any correspondence regarding the retraction of this article.

Signed: Jeremy Allen, Executive Editor, *Nanoscale Advances*.

Date: 17th July 2026.

**References**

1. J. Riaz, Y. Zhang, J. Cao, A. Bibi, M. Arif, Z. Zhang, D. Muhammad and X. Zhou, Facile synthesis of TiN nano sheets decorated Fe_2_O_3_ nanoparticles as novel cathode material for asymmetric supercapacitor, *Surf. Interfaces*, 2024, **46**, 104080, DOI: 10.1016/j.surfin.2024.104080.

